# Parked on the verge: vocational rehabilitation of long-term unemployed citizens – a mixed methods study

**DOI:** 10.1186/s13690-022-00838-x

**Published:** 2022-03-07

**Authors:** Lotte Nygaard Andersen, Mette Jensen Stochkendahl, Kirsten K. Roessler

**Affiliations:** 1 Department of Sports Science and Clinical Biomechanics, University of Southern Denmark, Odense, Denmark; 2 Chiropractic Knowledge Hub, Odense, Denmark; 3 Department of Psychology, University of Southern Denmark, Odense, Denmark

**Keywords:** Longitudinal survey, Interview, Social service interventions, Recovery of function

## Abstract

**Background:**

Vocational rehabilitation programs (VRP) developing and improving work ability are used in Denmark to assist long-term unemployed citizens with complex problems. The aims of this study were to (1) describe VRP-participants in relation to general health, well-being, work ability and self-efficacy at baseline and one-year follow-up, (2) obtain an understanding of VRP-participants’ personal development towards improving work ability, and (3) explore VRP-participants’ hopes and thoughts about their future.

**Methods:**

In a mixed methods approach, data from a longitudinal survey and semi-structured interviews were collected. In the quantitative longitudinal survey, all participants completed paper questionnaires at baseline and one-year follow-up. For the qualitative semi-structured interviews, VRP participants were recruited with a maximum variation sampling strategy through VRP coordinators and personal contact. Data were analysed by descriptive statistics and systematic text condensation. Following analysis, data were merged and presented in combination according to identified themes.

**Results:**

At baseline and one-year follow-up 146 (response rate 34%) and 74 participants (response rate 52%) respectively, responded to the questionnaire. Seven participants were interviewed. The analysis revealed four themes: 1) Individual explanations of life situation and health; 2) Finding the path; 3) Work as giving meaning to life; 4) Hope for the future. Despite self-reported scores indicating poor general health, lack of well-being, low work ability and low self-efficacy, VRP-activities seemed to have assisted participants in finding meaning in life. VRP-components that may be drivers of successful recovery processes were identified.

**Conclusions:**

VRP-participants experienced life situations that include multifactorial burdens, and low levels of general health, well-being, work ability, and self-efficacy at baseline and 1 year later. From the outset, most did not have a clear goal of employment, but over time, new goals were set as realistic opportunities for re-developing their work ability were explored. Successful core components of VRP were individually tailored programs and support, development of new relationships, and accommodated flexible internships and jobs.

**Trial registration:**

ClinicalTrials.gov Identifier: NCT02641704, date of registration December 29, 2015.

## Background

Unemployment is a key social determinant of health and health inequality [1–4]. Unemployment increases your risk of illness, disability [5] and poor mental health [6], and prolonged periods of unemployment are associated with higher all-cause mortality [7, 8]. The groups of unemployed who are most at risk of health loss are the long-term unemployed, the ageing [9], and those unemployed due to health reasons [3]. The relationship between poor health and unemployment is reciprocal [10]. Health problems like musculoskeletal pain, depressive mood and reduced sleep quality, and risk behaviours such as smoking and increased alcohol consumption predict prolonged unemployment [11], and people with disabilities have lower employment rates [12] and more difficulties in finding and keeping a job compared to people without such disease [13].

Re-employment of unemployed persons through health-improving interventions [14] or active labor market programs (which aims to develop specific work-related and job-seeking skills) hold important potential to reduce health inequalities, but the evidence of the effect of such programs is either limited [14] or the effects small [15, 16]. Generally, vocational rehabilitation programmes (VRP) consist of a combined therapeutic and work-related focus and have been widely used internationally to assist people experiencing complex health and/or life issues in preparing themselves or in regaining the ability to rejoin the workforce [17, 18]. VRP’s are typically designed with a focus on competitive employment to help people develop their chances of re-entering the workforce and may include individual placement and support activities [19, 20].

In Denmark, VRP’s are used to increase employment ability for unemployed citizens with complex health and social problems [21]. The Danish VRP-participants are long-term unemployed persons who have often been without a job for years or have never held a standard full-time job. The reasons for long-term unemployment are diverse and comprise both injury and illness; mental as well as physical. The economic compensation offered by the welfare state to unemployed is fairly comparable with other Nordic countries, and Swedish data indicate that the chance of getting a regular job or re-entering the workforce decrease with the length of the sickness absence before entry into a VRP [22]. Thus, the Danish VRP-participants are at risk of ending up on a permanent disability pension [21].

The Danish VRP’s are administered by the 98 municipalities in the public social service system and consist of individually tailored activities recommended by interdisciplinary rehabilitation teams [21]. Experience from the Netherlands shows that interdisciplinary re-employment programs can be effective in terms of physical and mental health improvements, but only among those who enter paid employment after participation in the program [23]. In Denmark, the effect of VRP has not yet been evaluated, however, a brief communication from the Danish Ministry of Employment [24] concluded that around 3% of the 20,818 persons who have completed VRP in Denmark in 2013–2019 ended up in a regular paid job, while 32% came closer to actively participating in the labor market (flexi-job, education or unemployment benefits). Half of the participants (52%) ended up on disability pension. These proportions are equivalent to data from Sweden where almost half of the participants in a VRP ended up on disability pension due to severe mental illness [22].

Current knowledge about VRP for vulnerable long-term unemployed is limited, and it is unclear which and how program components may bring the target group closer to work [25]. Due to the diversity of the unemployed population, it is unlikely that “one size fits all” and there is a need for a better understanding of population characteristics, the needs of the individuals, and how we best tailor VRP to their needs [25, 26]. Moving towards more knowledge on tailored program components entails incorporation of a range of methodologies to provide insights. Our study will be the first study in a Danish context based on a mixed methods approach (1). The approach with combination of quantitative and qualitative methods can provide more complete, detailed and in-depth descriptions of VRP participants than a single-stranded methodological approach. Results from this study adds knowledge about underlying processes and mechanisms involved in VRP leading to employment that has previously not been investigated (2).

This project aims to develop a detailed understanding of the health, psychosocial and work circumstances of VRP-participants, and of the important processes and mechanisms underlying the VRP-components [25, 27]. The objectives of this study were: (1) to describe the VRP-participants’ profile in relation to general health, well-being, work ability, and self-efficacy at baseline and at one-year follow-up, (2) to obtain an understanding of VRP-participants’ personal development towards improving work ability, and (3) to explore VRP-participants’ hopes and thoughts about their future.

## Methods

### Study design

A mixed methods exploratory approach [28] with the combined use of quantitative (a longitudinal survey) and qualitative (semi-structured interviews) data collection was used to investigate the study objectives. The mixed methods approach is useful when addressing complex issues and will allow more nuanced and broader insights than using a single method. Therefore, we assumed a pragmatic approach and considered multiple viewpoints, perspectives and positions to gain both a wider and deeper understanding of vocational rehabilitation in long-term unemployment [28]. The study is conducted in Sonderborg Municipality (1 out of 98 municipalities). Municipalities are individually responsible for VRP’s but a national reform [21, 29] defines the scheme on a general level for all municipalities for example establishment of rehabilitation team, assignment of coordinator and a frame for duration.

The results of the qualitative and quantitative data were presented in an embedded design. Both data types were collected concurrently, analyzed separately in the initial phase of analysis, and then merged and presented in combination according to themes identified and formulated in the qualitative analysis. The combined results were subsequently discussed and interpreted in the discussion [28].

This study was part of a larger project, and a complete description of methods used for the project has previously been described in Andersen et al. [27], and further findings will be reported in future publications.

### Participants

The participants were citizens enrolled in a VRP in Sonderborg Municipality, Denmark. Criteria for acceptance of participants into the VRP were defined by the Danish Ministry of Employment [21, 30]:

- being unemployed and aged 18 to 65 years
- being at high risk of permanent disability pension because of social, and/or health problems (mental and/or physical) that affect employability.
- not being ready to enter the labor market and requesting continued education or retraining of skills due the complex nature of their problems

Most of the VRP participants received social security benefits, which is the lowest level of economic compensation offered by the welfare state to long-term (more than 2 years) unemployed, while few received sickness or unemployment benefits (less than 2 years of unemployment.

### Vocational Rehabilitation Program (VRP)

A municipal rehabilitation team assigned participants to both the interdisciplinary VRP and a coordinator who supported and assisted the participant in carrying out the program. The municipal rehabilitation team determined the duration of the individual VRP (1-5 years) and tailored the activities, including one or more of [21, 31]:

- training in work and/or social skills (e.g. internship programs and leisure time activities)
- support service from the municipality (e.g. supporting contact person or health counselling)
- health-related self-management courses (e.g. in relation to anxiety or overweight)
- rehabilitative activities (physiotherapy or occupational therapy after diseases e.g. cancer or heart diseases)
- educational services (e.g. continuing education or school-leaving examination.

### Quantitative longitudinal survey procedures

To describe the participants’ profiles and assess any change over time, quantitative data were collected using standardized, validated questionnaires pertaining to the participants’ self-reported general health and well-being, work ability and self-efficacy at baseline and one-year follow-up.

In February 2016, a questionnaire was sent by mail to all participants who had been assigned to VRP during the previous year in Sonderborg Municipality. In addition, questionnaires were sent to all newly assigned participants during February 2016 to March 2017. Two weeks later, a telephone call was made to those who had not returned the questionnaire to remind them to return it. Four weeks after baseline, the questionnaire was re-sent to non-responders. One-year follow-up questionnaires with the same questions were sent from February 2017 until March 2018 to all participants who had returned the first questionnaire. If this was not returned, a second letter with the same questionnaire was sent.

#### Measures

*General health* was assessed using two selected items from the 36-Item Short Form Health Survey (SF-36) of self-perceived general health (“In general, would you say your health is” and “Compared to one year ago, how would you rate your health in general now?”). Answers were recorded using a 5-point Likert Scale ranging from” excellent” to” poor” and from” much better now than 1 year ago”, to” much worse than 1 year ago” [32, 33].

*Well-being* was assessed with the WHO-5 Well-Being Index [34, 35] measuring current mental well-being. The WHO-5 Well-Being Index is a 5-item questionnaire evaluating three domains: positive mood (good spirits, relaxation), vitality (being active and waking up fresh and rested), and general interest (being interested in things). Answers were recorded using a 6-point Likert Scale ranging from “all of the time” to “at no time” [36]. A WHO-5 raw score was calculated by totalling the five answers. The raw score was transformed to a percentage score (0–100%). Scores below 50 indicated major risk of depression [37].

*Work ability* was assessed with a single question from the Work Ability Index [38]. Studies have shown that the single item question is a reliable and easy tool with validity comparable with the full index [39]. The question was “Imagine that your work ability is worth 10 points when it is at its best. How many points would you give your present work ability?”. The answer was recorded on an 11-point numerical ration scale where 0 represents” not able to work” and 10 represents” the highest work ability” [39, 40].

*Self-efficacy* was assessed with the 10-item psychometric scale” The General Self-Efficacy Scale” [41]. It is designed to assess the belief in one’s competence to cope with a broad range of stressful or difficult demands in life [42]. Answers were recorded on a 4-point Likert Scale ranging from ‘not at all true’ to ‘exactly true’ [34, 41, 43]. All answers were added up to create a score ranging from 10 to 40 points.

#### Statistical analysis – part A

The quantitative data were analyzed using simple descriptive statistics. Continuous data were presented as median and interquartile ranges, and categorical data were presented as frequencies and proportions. Changes in work ability from baseline to follow-up were calculated as the percentage of participants improving or deteriorating and tested using McNemar’s test. A paired t-test was used to detect the mean change in WHO-5 and the General Self-Efficacy Scale.

Differences between responders and non-responders at follow-up were tested using a Chi2 test for categorical data and a paired t-test or the Wilcoxon rank-sum test depending on the distribution of continuous data. No data imputation was performed.

### Qualitative interview procedures

Semi-structured interviews [44] were conducted with seven participants. The sample consisted of five women and two men (Table 1). The interviews were conducted in Danish by the first author (LNA), who is an experienced interviewer, from September 2016 until August 2017.

**Table 1:** Interview participant characteristics

Synonym	Health problems	Civil status	Employment	VRP
Age group				Duration
Gender (w/m)				Activities
Anne 40–49 female	Mental	Married Children living at home	Manager	2.5 years so far Assigned 4 years Meetings with coordinator Course ‘Exercise and Motion’ Education Planned internship
Martin 40–49 male h	Musculoskeletal	Single	Chef Unskilled worker	1.5 years so far Assigned 2 years Meetings with coordinator Mentor visits 1/week Course ‘Exercise and Motion’ and ‘Coping with everyday life’
Birthe 50–59 female	Mental	Cohabiting Adult children	Skilled	1.5 years so far Assigned 3 years Meetings with coordinator Course ‘Exercise and Motion’ Internship
Jonna 50–59 female	Musculoskeletal	Married Adult children	Skilled	0.5–1 year so far - does not remember exactly Meetings with coordinator Course ‘Exercise and Motion’ Planned internship in a nursing home Applied for pension, refused
Allan 20–29 male	Mental	Single	Never had paid employment	Some months so far Assigned 3 years Meetings with coordinator Mentor meetings 2–3/week Psychologist consultations Substance abuse clinic consultations Course ‘Exercise and Motion’
Majken 30–39 female	Mental	Single Children living at home	Unskilled	2 years so far Assigned 3 years Meetings with coordinator Mentor meetings Relaxation therapist consultations Planned course ‘Exercise and Motion’ and planned psychiatric consultations Other: physiotherapy and psychology consultations (not VRP)
Karen 40–49 Female	Mental and musculoskeletal	Single Adult children	Skilled	1 year so far Assigned 5 years Meetings with coordinator Mentor meetings per phone or in person (customized) Relaxation therapist consultations Course ‘Exercise and Motion’

All participants’ names are pseudonyms

The recruitment strategy entailed enrolment of participants with varying characteristics in relation to gender, age, time into VRP, type of primary health problem, civil status, and previous employment. This maximum variation strategy allowed us to identify important shared patterns that cut across cases [45]. As recommended in qualitative studies, interview participants were recruited until we reached a rich variety in the study material that enabled an in-depth understanding of the study topic [46].

Six interview participants were recruited through their assigned coordinator, and one interview participant was contacted personally when the first author observed a VRP activity. We used a purposeful sampling strategy to recruit interview participants. This strategy is widely used in qualitative research and aims at recruiting participants who can provide in-depth information and nuanced insights about their VRP experience.

All interviews began with a briefing and ended with a de-briefing, where participants had the opportunity to ask questions if needed. Only the interviewer (author LNA) and the participant were present during the interview.

Depending on the individual preferences of the participants, the semi-structured interviews took place in either their private homes or in a room made available by the municipality at the social service office. Interviews lasted between 44 and 145 min and were transcribed verbatim. Subsequently, participants were invited to read and comment on the accuracy of the transcription. Two participants accepted this offer. Only one of the two participants had additional comments on the experience of financial hardship. None of the contacted potential interviewees declined to participate in an interview. Prior to the interview, participants were sent written information and ensured complete anonymity. The quotations presented in the results section were translated from Danish into English by a professional translator.

#### Interview guide

An interview guide with themes and related interview questions was formulated using everyday language [27]. The interview themes included: everyday life; the potential benefits of VRP; the development of work ability; relations between participant and coordinator, the impact of VRP, participants’ hopes and beliefs, their worries and anxiety. The interview guide allowed for a conversational two-way communication. Reflexive listening and clarifying questions were used when needed to enable in-depth descriptions from the interviewee [46].

#### Interview analysis

In the qualitative analysis, we used systematic text condensation [47]. The first author identified preliminary themes and discussed the content in the code groups and subgroups with co-authors. Dimensions of a recovery-oriented perspective [48] inspired the analysis as a framework; however, the analysis was not limited to the dimensions as an explorative approach was prioritized to not overlook important processes*,* concepts or ideas by using the chosen theoretical framework*.*

In addition to comparing results with previous literature, the recovery-oriented perspective [48] was used to discuss our findings, to develop in-depth understanding of the ways whereby participants can move on with their lives and grow despite their life circumstances. The conceptual recovery framework is developed based on studies of mental illness recovery and has been described as:

“…a deeply personal, unique process of changing one’s attitudes, values, feelings, goals, skills, and/or roles. It is a way of living a satisfying, hopeful, and contributing life even with limitations caused by illness. Recovery involves the development of new meaning and purpose in one’s life as one grows beyond the catastrophic effects of mental illness.” [48].

In this study the characteristics of the recovery journey is used. Many parallels can be drawn between mental illness recovery and the complex health situations the VPR participants were in; in particular, the journey towards moving on with their lives and grow as persons. In the coding process, we made sure that any data that fell outside of the coding frame were identified and examined to determine if important concepts or ideas were being missed by using the chosen theoretical framework.

Recovery is a multidimensional concept and various components tie it together. Key recovery processes are [49]:

1. *Connectedness* (receiving support from peers, groups and others; relationships and feeling inclusion as part of the community).
2. *Hope about the future* (encouraging belief in their potential; motivation to change; relationships generating hope; positive thinking and valuing success; dreams and aspirations.
3. *Identity* (rebuilding/redefining a positive sense of identity and overcoming stigma).
4. *Meaning in life* (discovering meaningfulness; identifying social roles and goals; focusing on quality of life and rebuilding life).
5. *Empowerment* (establishing personal responsibility and focusing on strengths).

## Results

Four interrelated themes emerged from the qualitative data analysis: 1) Individual explanations of life situation and health; 2) Finding the path; 3) Work as giving meaning to life; 4) Hopes for the future (subthemes: a) Hope for the work situation; b) Improvement in finances and its consequences – doing normal things). Themes 1) and 2) contain results from both qualitative and quantitative data, and themes 3 and 4 contain results from qualitative data only.

The baseline questionnaire was sent to 427 respondents (274 women, 146 men and seven gender not recorded). In total, 146 (34%) responded at baseline. Participants had a median age of 43.5 years, and 71% were female. For 37% of participants, the allocated program had an expected duration of more than 3 years. The median number of months in the VRP was 7.6. Descriptive characteristics of the participants at baseline are presented in Table 2.

**Table 2:** Participant baseline characteristics (*n* = 146)

Characteristic		
**Sex**, n (%)		
Female	104	(71.2)
Male	42	(28.8)
**Age** (years), median [IQR]*, *n* = 146	43.5	[31.0–50.0]
**Body Mass Index** (kg/m2), median [IQR], *n* = 137	27.1	[23.9–32.7]
**Number of months into the VRP** (months), median [IQR], *n* = 146	7.6	[1.2–14.0]
**Allocated duration of VRP**, n (%)		
1–2 years	39	(26.7)
2–3 years	50	(34.3)
3–4 years	8	(5.5)
4–5 years	46	(31.5)
**Civil status**, n (%)		
Married/cohabitant	78	(53.4)
Live alone	64	(43.8)
Other	3	(2.1)
**Education**, n (%)		
Less than 10 years of schooling	60	(41.1)
Basic vocational education (semi-skilled)	23	(15.8)
Full vocational education	25	(17.1)
Short higher education	10	(6.9)
Medium/long higher education	28	(19.2)
**Time since last paid employment**, n (%)		
Less than 1 year	1	(0.7)
1–2 years	9	(6.2)
2–3 years	12	(8.2)
More than 3 years	89	(61.0)
Never had paid employment	18	(12.3)

*VRP* Vocational Rehabilitation Program, *IQR* Interquartile Range

At follow-up, 74 (51%) responded to the questionnaire (corresponding to 17% of individuals who received the baseline questionnaire). Respondents were older than non-respondents (mean difference of 3.9 years, *p* = 0.04). There was no statistically significant difference at baseline between follow-up respondents and non-respondents regarding gender, time in the program, allocated time, BMI, marital status and educational level. There were also no statistically significant differences at baseline regarding general health, work ability, well-being or self-efficacy.

### Individual explanations of life situation and health

In general, the respondents scored low both on self-reported general health and well-being (Table 3) at baseline and follow up. At baseline, 37% of the participants rated their general health as *poor* and 39.7% rated it as *fair.* Among those who completed the follow-up questionnaire, the general health and well-being of ten respondents (13.5%) deteriorated, 48 (64.9%) were unchanged, and 15 (20.3%) improved. At follow-up, the participants’ ratings of their general health compared to 1 year ago showed that only a small proportion rated their health as improved.

**Table 3:** General health, well-being, work ability, and self-efficacy at baseline and follow-up

	Baseline ***n*** = 146		Follow up ***n*** = 74		Change ***n*** = 74			
**General health**, n (%)					Deteriorated, n (%)		Improved, n (%)	
Excellent	1	(0.7)	1	(1.4)	10 (13.5)		15 (20.3)	
Very good	13	(8.9)	5	(6.8)				
Good	19	(13.0)	17	(23.0)				
Fair	58	(39.7)	23	(31.1)				
Poor	54	(37.0)	27	(36.5)				
Missing	1	(0.7)	1	(1.4)				
**General health, compared to 1 year ago**, n (%)								
Much better than 1 year ago			7	(9.5)				
Somewhat better than 1 year ago			11	(14.8)				
About the same			25	(33.8)				
Somewhat worse than 1 year ago			17	(23.0)				
Much worse than 1 year ago			13	(17.6)				
Missing			1	(1.4)				
					Mean change (SD)		p*	
**WHO-5 Well-being Index**, median [IQR] (*n* = 70)	28	[16–52]	32	[20–56]	3.73	(±17.67)	0.08	
**Work Ability Score**, median [IQR], (*n* = 72)	1	[0–2]	1	[0–3]	0.35	(±1.24)	0.33	
**General Self-Efficacy Scale**, median [IQR] (*n* = 69)	20	[15–26]	21	[16.5–7.5]	1.13	(±5.5)	0.09	

*n* number, *IQR* Interquartile range, *SD* standard deviation, *WHO* World Health Organization

*The change in Work Ability Score is calculated using McNemar’s test; WHO-5 and General Self-Efficacy Scale changes are calculated using paired t-test

A score lower than 50 in the baseline values of the WHO-5 Well-being Index, indicated that participants were at a significant risk of depression and stress conditions. A small positive tendency towards a higher score at follow-up was seen (mean difference of 3.73 (±17.67)), though the change was not statistically significant (*p* = 0.08) (Table 3).

The poor self-reported health was mirrored in the participant interviews. A commonly described feeling was that of ‘not feeling well’. The participants described multiple factors involving physical, psychological and social aspects that led to their life situations and inability to work.

For some, inability to work was attributed to health problems occurring suddenly late in life, which led to a general deterioration in their physical and mental health. One participant, Martin, described being involved in a traffic accident causing symptoms of whiplash several years previously, but he managed to return to work after 6 months. However, over the years that followed he experienced stress reactions and he said that in a way he lost faith in his ability to recover: ‘*My health has started to decline (…) so there’s nothing I can do about it.’*

Work-related stress was also given as explanation for inability to work. One participant described how she was caught in the system:

My doctor found that it was probably something more like a depression, that I had burned out. The only thing I could do was, in fact, to exist. There was no juice in my bones, it was just some kind of lump. I didn’t understand that your body could get ill when your spirit is completely crushed, so I took sick leave again and I just got more and more stuck in the system. (Birthe)

Other participants had never entered the labor market because of early life circumstances. Allan described how several deaths among his closest relatives had led to personal substance abuse, because the drugs community was an opening for him to be with other people: ‘*So it became drugs, because then I was together with other people and so I had someone to talk to’*.

For Majken, too, her birth and upbringing were factors that created complications later in life, including her entry to the labor market. She suffered from post-traumatic stress because of challenges both in youth and adult life, and she struggled with substance abuse:

My biological mother was a junkie (…), I was always stressed and my foster mum was an alcoholic and was a diagnosed schizophrenic. I’ve been an addict myself, did heroin, too, and lived on the street. (Majken)

### Finding the path

At baseline, many survey respondents reported that they had not been in paid employment for several years, 61% had not had a job in more than 3 years, and 12% had never had a job (Table 2). Generally, they rated their work ability as very low with median scores of 1 at both baseline and follow-up. Likewise, the respondents had low scores of 20 and 21 at baseline and follow-up, respectively, on the General Self-Efficacy Scale (mean change of 1.1, *p* = 0.09) (Table 3).

In the interviews, participants described the VRP as a step towards clarifying, finding or developing realistic goals relating to working life. While some participants had had no experience of working life, others needed to recreate goals after they had lost meaningful jobs: *‘After I was sacked, I thought is there anything I can do? I can’t do the thing I enjoy most of all’* (Birthe).

Though most participants described a clear desire to work, not all of them had clear goals when starting VRP. However, they did develop more specific goals over time. When Anne started VRP, she had no job-related goals, though she looked favorably at the process of setting goals for the future. Anne found that her coordinator gave her positive, individual support in the goal-setting process:

I was paddling round on a wide-open lake; I had no idea in which direction to go. So, for me it was all about, yeah, being kept a bit in place (by the coordinator), at least in a lake, you know, I wasn’t out on the open sea. (Anne)

Martin described a clear goal of getting a job and a longing for a stable and predictable everyday life. His personal goal clearly reflected an aversion to being dependent on the social service system and VRP: *‘It’s to get out of this system and then get a job, that’s much better than this’*. (Martin).

All participants except one were motivated to building up their work ability, to be in an internship or go to work. Jonna was alone in not sharing this feeling of being motivated to be in an internship:

If you can feel that it’s not leading anywhere, what’s the point? What on earth do you think it costs to have me on their books? (Jonna)

Jonna had already been in the labor market for many years, had experienced substantial physical limitations and was the oldest of the interviewees. She described how she had once been motivated to have a work life, but not anymore. In contrast to Jonna, Karen explained how her physical condition had improved through participation in VRP-activities led by physiotherapists:

You get more self-confidence when you get some experiences of success. When I started, I couldn’t do anything, I couldn’t lift my arm, I couldn’t even watch a film on the tv, because I couldn’t lift my head, I used to walk around like this (shows bowed head). I am in a bit better shape now, happier (…) you feel that you’ve done something, even though it’s only an hour. I know that people work eight hours every day, but this is what I have to do, and I’ve been good at it. (Karen)

Karen went on to describe, how she had succeeded with various activities when she participated on her own terms, how this had been important for the development of her ability to take part in valued social activities, and how this had helped her develop a positive self-image. Previously, she would typically withdraw from social situations *‘I had hundreds of excuses for why I couldn’t do that and it was probably best if I stayed home’*, but now she described that the VRP process impacted positively on her ability to socialize.

### Work as giving meaning to life

Almost all interviewed participants explained how not having a job and receiving welfare payment were accompanied by feelings of contributing to society in a meaningful way. Before Anne was assigned to VRP, she was unable to work because of her mental health problems. She did not receive welfare payment, and her husband was the sole breadwinner of the family:*‘I couldn’t contribute, but then I didn’t take anything either (…) like being in no-man’s land’*. Anne felt that the assignment to VRP made her a part of society, because she got help, and society showed an interest in her. She had arranged meetings with the coordinator, who supported her in taking the next step and developing employability.

Some of the participants described their motivation for work and internships with words that indicated that having a job is important. Martin described how his motivation for a job was not related to the content of the work but to the feeling of having and managing a job and to the work environment:

It can be something totally brain dead (…), a good working environment, that’s the main thing for me. Wages and tasks that’s kind of in the background, they aren’t that important. It has to be something I can cope with. (Martin)

Birthe stressed the way the friendly working environment served as a vehicle for making her feel useful.

To be met by colleagues, it’s great to meet someone and they say hi! Having someone besides what’s at home, that gives me a happy feeling (…) I was a bit anxious, whether I’d manage, but it is just so great that I can do something. (Birthe)

At the same time, she pinpoints the crucial importance of being accepted among colleagues on her own terms. A colleague once confronted Birthe with the fact that she only worked few hours a week in the internship. Afterwards, she reflected on her own and her colleagues’ acceptance of her situation with working 2 h a day:

That’s a brilliant life. No, it sodding well isn’t! I’d a hundred times rather have their life than my life, so it makes me so bloody sad, that sort of thing. (Birthe)

Another aspect of having a job is that it offers the opportunity to go on an annual holiday. Several participants described how this possibility supported their notion of playing a part in society, and thereby also feeling like a normal person themselves:

It was a huge thing, being able to say that I was going on holiday. Because you might be on income support, but you don’t get a holiday, you’re not off work. I take this thing (VRP) as a kind of job, ‘cos you’ve got something you have to do and now I shan’t be coming for a while because I am ‘off’, and you know you’re going to be starting again. (Karen)

Other participants interviewed also elaborated on the significance of going to work, explaining that the sense of being useful and of others appreciating their work are feelings they long for*.*

### Hope for the future

#### Hope for the work situation

Regarding her overall life situation, Anne emphasized the importance of continuing to believe in and hope for a change for the better *‘that’s the most important thing, keeping on believing and hoping and learning to understand’.* The interviewed participants had their hope of re-establishing work ability and (re-)joining the workforce in common. Birthe described how she hoped her situation would look like in 3 years: *‘I certainly hope that I have a flexi-job that I really like, to the extent that I can cope with it’*.

Participants expressed positive feelings related to having an employment at all. Most participants clearly expressed hopes for flexi-jobs (working on special terms designed for people with permanently reduced ability to work), but they were aware that their condition required individually tailored jobs. Birthe elaborated on her hopes in the contexts of her fear that her VRP would be extended:

That’s really my biggest fear (…). There are so many things that is normal for ordinary people to do, but which we are not part of, but I feel as though I’m just parked here on the verge. (Birthe)

#### Improvement in finances and its consequences – doing normal things

Interview participants described how they longed for what they called “doing normal things”, but they felt that their personal financial situation was a constraining factor to the point where they even believed that the lack of financial resources was restricting them from moving on and developing their ability to work.

Sometimes, when they have said things to me like ‘there’s loads of time, how long was it you were going to be on a VRP, three years?’. When I feel like, well, I’d just like to get it over with, because then I will hopefully be able to get a flexi-job, so I can get paid a salary, which might help me just a little bit, so I can get to be just like everyone else again. (Birthe)

Martin explained that his physical condition had previously benefitted from warm water pool therapy and physiotherapy, but his financial situation did not allow him to pay for therapy: “They suggested to me that I should go to some physio (…), I just don’t have the money to pay for it”.

Martin did not doubt the personal value of having a job, but it was clear that the financial benefit was also important. Participants accepted that their financial situation would probably never be as good as when they had a regular job, but it was clear that they longed for what they called a normal life. For the participants, a normal life included some level of independence, even though their situation has forced them to adopt new values and priorities, as clearly expressed by Birthe:

I’ve become really good at going to charity shops and I’ll keep doing that. I’ve hardly got any clothes that aren’t from a charity shop. (Birthe)

## Discussion

In a mixed methods design, we analysed data from 146 participants in a VRP in Denmark responding to a mail survey at baseline and one-year follow-up, and seven face-to-face semi-structured interviews. The participants were assigned to VRP due to unemployment and being at high-risk of permanent disability pension because of social, and/or health problems (mental and/or physical) that affect employability. They are not ready to enter the labor market and they are at a high risk of permanent disability pension. The survey indicated overall poor self-reported health, well-being and work ability at both baseline and follow-up, which were reflected in the individual life stories of the participants. Four themes emerged from the interviews: 1) Individual explanations of life situation and health; 2) Finding the path; 3) Work as giving meaning to life; and 4) Hope for the future. The themes identified important VRP-components that may be drivers of successful recovery processes. These emphasize the importance of internships and workplaces that can accommodate the participants and their functional limitation and their (lack of) previous work experience.

### Participants’ profiles - needs for recovery assistance and development

#### General health and well-being

Generally, the participants rated their general health and well-being as relatively low at both baseline and follow-up and dual burdening situations including e.g. substance abuse and mental illness were described by interview participants [50]. Further, lack of hope and motivation for a future working life were described in one of the interviews and also mirrored by the low well-being scores. Our findings may mirror those of previous studies suggesting that the participant’s poor health situation and negative feelings combined with being close to retirement age may have contributed to the participant’s feeling that recovery for a life of work was without meaning [51, 52]. Prior studies have also pointed towards severity of symptoms [52] and age [3] as important to the ability to work. That emphasizes the importance of considering the age, general health and well-being of the participants as potential determinants of recovery when the aim is to assist recovery and development towards employability of long-term unemployed [3]. It also stresses the need for recovery from not only one, but all the conditions from which the participants suffer [50].

#### Work ability and self-efficacy

Overall, the participants reported very low work ability at both baseline and follow-up with a median of 1 at both data collection points. In comparison, Danish workers on sick-leave due to musculoskeletal pain reported work ability scores ranging from 2.5 to 3.1 [53], and a large cohort study of blue-collar workers from various occupational groups showed a mean of 8.4 [54]. A very low work ability has been associated with an increased risk of disability pension [55], which renders the participants in our study at an increased risk of leaving the active labor force. Self-efficacy is a prerequisite for the ability to perform valued activities, also in work-related contexts [42]. In this study, self-efficacy scores were low at both survey time points whereas the qualitative results indicated positive developments. To change the trajectory towards disability, the participants would need to improve their work ability through increased self-efficacy and define new working life goals [40]. The interviews highlighted that participants were at different stages of finding these goals related to working life, but also that VRP supported participants in finding goals related to improving work ability, and in that way, VRP gave the participants opportunity to increase their self-efficacy.

### VRP – aiming towards recovery

The individual recovery processes identified in VRP for developing work ability are here summarized in the *recovery concept’s five key processes* [48, 49, 51, 56]:

1. Referral to internships and/or workplaces with flexible demands gave opportunities for successful work, *feelings of connectedness to others*, a sense of being part of the community and a degree of financial independence;
2. Assistance in positive thinking to stimulate *hope about the future* from a coordinator or from VRP courses was significant for setting goals;
3. Building-up general health positively affected self-efficacy and thereby a *positive sense of identity*, enabling participants to do valued things;
4. Making room for individual processes of *re-finding meaning in life,* for example by accepting job opportunities;
5. Building success upon success and overcoming fear promote occupational *empowerment*

The key recovery processes identified here facilitated participants’ development of work ability in an interrelated manner.

Having a job, being in internships or taking part in VRP-activities were valued by the participants because it helped them feel a part of society and gave a sense of being connected to others [57]. This feeling of being connected with others and developing of new relationships with colleagues, customers, the VPR-coordinator or other municipal officials were important for the recovery process [49]. Partaking in VRP-activities and the contact with the coordinator provided the participants with new relationships that may potentially have stimulated the participants’ hope, supported the goal-setting process and assisted the participants’ development of new meaning and purpose in their life [49, 58]. On the road to recovery, it is necessary for the participants to not only accept their individual conditions, but also to accept that their situation as such has not changed [49]. In the interviews the participants described acceptance and how they were changing in order to discover new goals and meaning. The participants’ close relations must also accept them as they are and the journey they have embarked on. If they are not accepted, the participants will not feel connected to others and will develop or maintain a negative sense of identity, and thereby not become empowered [49, 58]. Loss of self-esteem and feelings of stigma [48] are therefore important barriers to developing work ability and developing a positive sense of identity. The stigma was exemplified in the accounts of how the participants felt like being part of the community when as employees, they could legitimately take annual holiday leave and how having a job gave them the financial opportunity to do things that they believed normal people did [59]. Especially individually tailored internships and VRP-activities aiming at increasing socializing abilities were valued by the participants and were potentially core components in the development of the participants’ positive sense of identity. When on VRP internships, participants felt empowered to overcome their fear of not being able to cope with a job again [49]. However, it has previously been shown to be important for empowerment and success in occupational settings that the internships, workplaces and other VRP-activities are flexible and adaptable to individual values and preferences [58, 59].

Supported employment and individual placement initiatives have been shown to be effective in helping people with mental illness find competitive and sustainable employment [17, 60]. All interviewed participants except one made clear their hopes of getting individually designed jobs, and they acknowledged that VRP was a way to realize their hopes.

The recovery process was perceived as time-consuming and the participants experienced the time frame as being long. For example, building up the physical capacity was described by participants as taking time, despite participants having strong hopes of a quick recovery [48]. Previously Strickler et al. [52] have shown, that a long time frame for employment recovery is profitable. Therefore, tailoring internships and VRP-activities according to realistic time frames are important to the recovery process and must be acknowledged when building up work ability in VRP.

### Strengths and limitations

The major strength of this study is the combined use of qualitative and quantitative methodologies and integrated analysis to explore the experiences of the participants. This allows for both in-depth knowledge of the participants’ experiences and perspectives, and width of our findings. The results provide important insights into the study population and the potentially important VRP components and mechanisms on which future programs can be build.

We used a maximum variation sampling strategy and a gatekeeper for the recruitment of interview participants. While the gatekeeper was instructed to reach out to a variety of potential interview participants, we cannot exclude that the gatekeeper did not reach the most vulnerable and, thus, we did not capture their voices. We included seven participants in the interview part of the study, and the data collection stopped when we reached in-depth information about participants’ experiences and a rich variety in the collected data material. Our aim was not generalised answers in the qualitative part of the study [5, 6].

Thus the interviews provided ample and rich information about the rehabilitation process and the participation in VRP from the participants’ perspective [44]. The aim of the qualitative part of the study was not to arrive at an objective or generalised truth [44], but to gain in-depth understanding of the issues. The results indicate that older age may impact hope and motivation for future working life, but the data does not allow us to investigate this further, and future research should elucidate the impact of age on VRP effect.

. Furthermore it is a strength that results have been linked to theoretical concepts using recovery as theoretical framework, it enables transferability of the results to other contexts [61]. However, we sampled participants from one municipality only. Danish governmental reports indicate a wide variety in the use and content of VRP across municipalities, and therefore caution should be taken when generalising our findings to other municipalities.

In the quantitative part, validated questionnaires were used to describe participants’ characteristics at two different time points, however the study was not designed or powered to evaluate the effect of the program. Only around 50% of participants at baseline returned the one-year follow-up questionnaire with respondents being older. This may hamper the generalizability of our survey findings, but arguments for acceptable response rates from 25% have been put forward [62, 63]. There are several factors that may explain the loss of survey participants to follow-up [64]. These include the health status and income of the participants, the length of the questionnaire and length of follow-up period. The overall low recruitment and retention rate probably reflect the difficult life situations VRP-participants find themselves in (and in which partaking in research is not a priority), and the low level of personal resources and capacity for unfamiliar or extra activities. Similarly to the interviews, it is likely that we have included the highest functioning participants with the least amount of problems. Conversely, it is possible that the included participants may be individuals wishing to have their voices heard, as well as it being possible that some of the baseline respondents had left the VRP at the time of follow-up due to finding work. Given the low health and work ability scores and the burdened life stories, this only emphasizes the level of vulnerability and social marginalization this group of long-term unemployed experience.

Our study adds to the knowledge base about mechanisms and processes underlying possible effects of VRP. Our results add detailed understanding of the health, psychosocial and work circumstances of VRP-participants, and results will be valuable for the design of future research-based effect evaluations of the VRP in Denmark Nonetheless, more research is needed in a Danish context, and a formal evaluation of VRPs in Denmark is still to be conducted.

## Conclusions

The participants in VRP experienced difficult and diverse life situations that include multiple physical, mental and social problems, which manifested as low levels of general health, well-being, work ability, and self-efficacy at both baseline and 1 year later. From the outset, most did not have a clear goal of employment, but over time, new goals were set as realistic opportunities for re-developing their work ability were explored. The core components of VRP facilitating the participants’ personal development towards improving work ability were individual support, meaningful relationships with a VRP coordinator, empowerment, and self-perceived improvements in physical and mental well-being. Engaging in accommodated internships gave the participants an opportunity to gain successful work experience, become empowered, develop new relationships, and a positive sense of identity, which was essential for a meaningful life. The participants longed for “normality” and change for the better through work (re-)entry, but the participants’ health problems and life situations negatively affected their hope for re-entering the workforce. Thus, the recovery process was perceived as long and time consuming, but also worthwhile.

Our findings highlight the need for individually designed VRP-activities, including individual support and flexible jobs or internships that take into consideration all physical and mental health barriers experienced by the individual. Tailoring internships and VRP-activities according to realistic time frames are important to the recovery process and must be acknowledged when building up work ability in VRP.

In the future it may be worth considering whether it would be beneficial to put even more emphasis on the need for individual tailoring and the participants’ general health situation in VRP as determinants for participants’ chances of recovery.

## Data Availability

The datasets are not publicly accessible in order to preserve the privacy of participants. However, they are available from the corresponding author on reasonable request.

## Abbreviations

IQR: Interquartile rang
n: Number
SD: Standard deviation
VRP: Vocational rehabilitation program
WHO: World Health Organization
